# Silibinin Attenuates Sulfur Mustard Analog-Induced Skin Injury by Targeting Multiple Pathways Connecting Oxidative Stress and Inflammation

**DOI:** 10.1371/journal.pone.0046149

**Published:** 2012-09-27

**Authors:** Neera Tewari-Singh, Anil K. Jain, Swetha Inturi, Chapla Agarwal, Carl W. White, Rajesh Agarwal

**Affiliations:** 1 Department of Pharmaceutical Sciences, University of Colorado Denver, Aurora, Colorado, United States of America; 2 Department of Pediatrics, University of Colorado Denver, Aurora, Colorado, United States of America; National Institutes of Health, United States of America

## Abstract

Chemical warfare agent sulfur mustard (HD) inflicts delayed blistering and incapacitating skin injuries. To identify effective countermeasures against HD-induced skin injuries, efficacy studies were carried out employing HD analog 2-chloroethyl ethyl sulfide (CEES)-induced injury biomarkers in skin cells and SKH-1 hairless mouse skin. The data demonstrate strong therapeutic efficacy of silibinin, a natural flavanone, in attenuating CEES-induced skin injury and oxidative stress. In skin cells, silibinin (10 µM) treatment 30 min after 0.35/0.5 mM CEES exposure caused a significant (p<0.05) reversal in CEES-induced decrease in cell viability, apoptotic and necrotic cell death, DNA damage, and an increase in oxidative stress. Silibinin (1 mg) applied topically to mouse skin 30 min post-CEES exposure (2 mg), was effective in reversing CEES-induced increases in skin bi-fold (62%) and epidermal thickness (85%), apoptotic cell death (70%), myeloperoxidase activity (complete reversal), induction of iNOS, COX-2, and MMP-9 protein levels (>90%), and activation of transcription factors NF-κB and AP-1 (complete reversal). Similarly, silibinin treatment was also effective in attenuating CEES-induced oxidative stress measured by 4-hydroxynonenal and 5,5-dimethyl-2-(8-octanoic acid)-1-pyrolline N-oxide protein adduct formation, and 8-oxo-2-deoxyguanosine levels. Since our previous studies implicated oxidative stress, in part, in CEES-induced toxic responses, the reversal of CEES-induced oxidative stress and other toxic effects by silibinin in this study indicate its pleiotropic therapeutic efficacy. Together, these findings support further optimization of silibinin in HD skin toxicity model to develop a novel effective therapy for skin injuries by vesicants.

## Introduction

Skin injuries inflicted by vesicating chemical warfare agent sulfur mustard [HD, bis(2-chloroethyl) sulfide)] can be acute, excruciating and last for several years [1]–[4]. HD exerts a delayed inflammatory response and cytotoxicity in the basal keratinocytes leading to epidermal-dermal separation and blister formation [5]–[9]. HD is a bifunctional alkylating agent that reacts with cellular targets including lipids, proteins and DNA, and mechanistic aspects of HD-induced skin injuries include oxidative stress, DNA damage and cell cycle pathways, caspase and poly (ADP–ribose) polymerases (PARP), mitogen activated protein kinases (MAPKs) and Akt pathways, transcription factors activator protein-1 (AP-1) and nuclear factor- κB (NF-κB), matrix metalloprotease-9 (MMP-9), inflammatory mediators cyclooxygenase-2 (COX-2), inducible nitric oxide synthase (iNOS), cytokines, and calcium signaling [5], [6], [10]–[17]. The treatment strategies so far for HD-induced skin injuries have been symptomatic and do not target the multiple pathways of insult, and therefore, could not be developed as effective therapeutics. Agents that can target HD-induced oxidative stress are important therapeutic options because oxidative stress is reported as an immediate key consequence of HD exposure, which can lead to the activation of intricate signaling pathways [14], [18]. Several antioxidants such as GSH, N-acetyl cysteine (NAC), sulforaphane, zinc oxide, zinc chloride, butylated hydroxyanisole (BHA), ebselen, desferrioxamine, L-thiocitrulline (l-TC) have shown beneficial effects in reducing vesicant-induced skin injuries [14], [18], [19]; however, most of them exhibit stronger protective effect than therapeutic potential [14], [18]–[20]. Accordingly, in this study, we focused our efforts on the identification of effective mechanism-based pleiotropic or multifunctional rescue therapy to target the complex pathways triggered by vesicant exposure that lead to incapacitating skin injuries.

**Figure 1:**
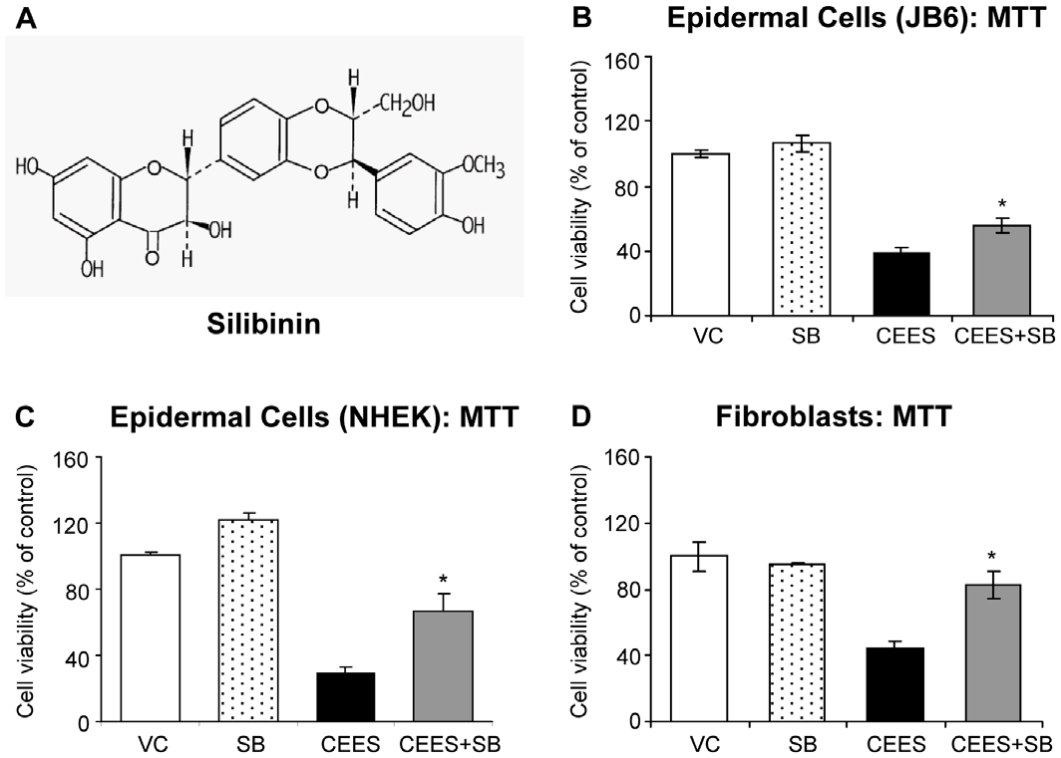
Silibinin reverses CEES-induced decrease in cell viability in skin cells. Chemical structure of silibinin, a naturally occurring flavonolignan (**A**). Mouse epidermal JB6 cells (**B**), normal human epidermal keratinocytes (NHEK; **C**), and mouse fibroblasts (**D**) were seeded (2000 cells/well) in 96 well plates and grown overnight. The cells were then exposed to DMSO or 0.35 mM CEES in DMSO, or treated with either 10 µM silibinin alone or with silibinin following 30 min CEES exposure for 24 h (JB6 cells and fibroblasts) and 48 h (NHEK) as detailed under Materials and Methods. Thereafter, MTT was added to the cells for 4 h at 37°C and absorbance was read at 540 nm following the addition of DMSO as detailed under Materials and Methods. Blank control readings were subtracted from all the sample readings taken. MTT assay data shown are mean ± SEM of 3–6 independent samples for each treatment. *, p<0.05 as compared to CEES exposed group. VC, vehicle (DMSO) control; SB, silibinin; CEES+SB, silibinin treatment 30 min after CEES exposure.

Silibinin (C_25_H_22_O_10_; Fig. 1A), a non-toxic, naturally occurring flavanone isolated from the seeds of Milk Thistle [*Silybum marianum* (L.)] has been used as a traditional medicine for years to treat various liver disorders and is sold as a dietary supplement around the world including the United States [21]–[23]. Silibinin possesses strong antioxidant, anti-inflammatory, anti-cancer and cancer chemopreventive properties, and this drug is in clinical trials for its efficacy against several cancers [22], [24]. Since it is reported that silibinin targets multiple signaling pathways including oxidative stress and inflammation to prevent skin injuries and cancer by genotoxic and other agents that are similar to the pathways triggered following vesicant exposure [22], [25]–[28], we hypothesize that silibinin would exert strong efficacy in attenuating HD and other vesicant-induced skin injuries. Consequently, efficacy studies with silibinin were carried out employing HD analog 2-chloroethyl ethyl sulfide (CEES)-induced injury biomarkers established from our earlier studies in skin cells and SKH-1 hairless mouse skin [2], [6], [9], [12], [13], [29]. Findings herein show that silibinin is an effective therapeutic agent that attenuates CEES-induced injury including oxidative stress in the skin cells and mouse skin tissue, suggesting its strong potential as novel treatment for skin injuries by vesicants.

## Materials and Methods

### Culture and treatment of cells

JB6 cells (American Type Culture Collection; ATCC; Manassas, VA) and fibroblasts (isolated from neonatal SKH-1 hairless mice skin as per Hakkinen et al. [30] were cultured as described earlier [6], [29]. Briefly, JB6 cells were cultured in minimal essential medium (MEM; Gibco BRL, Grand Island, NY) containing 5% heat-inactivated fetal bovine serum (FBS) and 25 µg/mL gentamycin. Fibroblasts were cultured in Dulbecco's modified Eagle's medium (DMEM) with 10% FBS, 25 mM HEPES and 1% Antibiotic and Antimycotic (Gibco BRL). Normal human epidermal keratinocytes (NHEK) were obtained from Lonza (Walkersville, MD), and cultured according to the instructions in the Keratinocyte Growth Medium (KGM) with provided additives. Cells grown over night (O/N) under standard conditions were either exposed to dimethyl sulfoxide (DMSO), or CEES (0.35/0.5 mM; Sigma-Aldrich Chemical Co. St. Louis, MO) in DMSO, or treated with 10 µM silibinin (Sigma-Aldrich Chemical Co.) in DMSO following 30 min CEES exposure, and studies were carried out at the desired time points of up to 48 h. The final concentration of DMSO in the culture medium during treatments did not exceed 0.1% (v/v). All CEES preparations and treatments were carried out under a safety laminar hood using all required and approved personal protective equipment [2].

### Measurement of cell viability and quantification of apoptotic and necrotic cell death

MTT assay (with 1 mg/mL of MTT; Sigma-Aldrich Chemical Co) for cell viability was conducted, as described earlier, at 24 or 48 h following the desired exposures and treatment of cells [6]. Briefly, at the desired time point the culture medium was removed, MTT was added to the cells in serum free medium for 4 h at 37°C. The MTT solution was then removed and absorbance was read at 540 nm following the addition of 100 µL DMSO. Hoechst-propidium iodide (PI) staining, was carried out after 24 h of above mentioned exposures and treatment by staining cells with 10 µL of PI (1 mg/mL) and Hoechst 33342 (1 mg/mL; Sigma chemical Co) at a ratio of 3∶1, as reported earlier. Briefly, at the end of each treatment time, both floaters and attached cells were collected, washed twice with 1× PBS, kept on ice and stained with Hoechst-PI dye at a ratio of 3∶1. For Hoechst 33342, the excitation wavelength is 350 nm and emission at 461 nm and for PI excitation is at 535 nm and emission at 617 nm. Quantification of cells was performed in triplicate for each treatment and 200 cells per sample were counted in different fields (at least 10 random fields per sample) to score for percent live, apoptotic and necrotic cells as reported earlier [6], using a fluorescent microscope (Zeiss Axioscope-2 plus-HBO100, Zeiss, Jena, Germany. The cells were classified as follows: (i) live cells-blue fluorescence and normal structure (Hoechst stained), (ii) early apoptotic cells- bright blue florescence and enlarged with condensed, or fragmented chromatin (Hoechst stained), (iii) late apoptotic cells- bright red (pink to white) fluorescence in the centre due to condensed chromatin (Hoechst stained) and (iv) necrotic cells-bright red fluorescence and enlarged (PI stained).

### Comet assay

DNA damage was measured using single cell gel electrophoresis (SCGE) or alkaline comet assay (pH≥13) and staining with 3 µg/mL of PI as reported recently [29]. In brief, cell suspension (300 µL) from the exposed and treatment groups after 1 h was mixed with 1% low-melting point agarose and was added to slides precoated with 1% normal-melting point agarose. These slides were then incubated O/N at 4°C in lysis solution, washed, left for DNA unwinding and subjected to electrophoresis for 20 min at 22 V and 200 mA. Next, slides were neutralized in 500 mM Tris-HCl, pH 8.0, washed with ddH_2_O and dried O/N after staining with 3 µg/mL of PI. Fluorescence of the DNA in cells and in comets seen in case of DNA damage was scored using a Nikon invert microscope (Nikon Eclipse TE300) at ×200 magnification, and images were captured using an attached CoolSNAP_ES_ CCD camera. One hundred and fifty cells, 50 each on triplicate slides were captured and tail extent moment (TEM; product of tail length and percentage tail DNA) scored using Komet 5.5 software (ANDOR Technology, South Windsor, CT).

### Cellular and mitochondrial superoxide detection

Following the desired exposures and treatment for 4–6 h, cellular and mitochondrial ROS, mainly superoxide (O_2_^−^) were measured in cells employing 5 µM cellular O_2_^−^ indicator dihydroethidium (DHE) and mitochondrial O_2_^−^ indicator MitoSOX Red (Invitrogen; Carlsbad, CA), respectively, and positive controls included as detailed earlier [29]. Briefly, cells, treated with 30 µM antimycin A for 20 min or 200 nM valinomycin for 1 h for the selectivity of MitoSOX red and DHE staining, respectively. In our previous studies, 100 µM H_2_O_2_ treatment was used as a negative control [29]. The cells were incubated for 30 min or 1 h with DHE or MitoSOX Red, respectively. Thereafter, cells were washed with 1× PBS, scraped and collected in microtiter tubes and the live cell fluorescence was determined using flowcytometry. Fluorescence was measured at the core services of the University Of Colorado Cancer Center.

### Animal treatment

Female SKH-1 hairless mice (4 to 5 weeks of age) were purchased from Charles River Laboratories (Wilmington, MA), housed and acclimatized under standard conditions at the Center of Laboratory Animal Care, and studies were carried out according to the specified protocol approved by the Institutional Animal Care and Use Committee (IACUC) of the University of Colorado Denver. All the CEES exposures were carried out following the prepared Standard Operating Procedure (SOP) by the IACUC of the University of Colorado Denver, animals were monitored every day to record any abnormal changes in their health or behavior and veterinarian consulted whenever required. Mice (5 per group) were untreated, exposed to either 200 µL acetone alone, 2 mg (80 mg/kg) CEES in 200 µL acetone, silibinin alone or treated with 0.5 or 1 mg (20 or 40 mg/kg) silibinin in acetone after 30 min of CEES exposure. Following the above mentioned exposures and treatments for 24 h, mice were euthanized, the dorsal skin was collected and either snap frozen in liquid nitrogen or fixed in 10% phosphate buffered formalin as detailed earlier [2].

### Measurement of skin bi-fold thickness, epidermal thickness and quantification of apoptotic cells

Using an electronic digital caliper (Marathon Inc. Belleville, ON, Canada), the dorsal skin bi-fold thickness of mice was measured (mm) at 9, 12 and 24 h following the desired exposures and treatments. Following tissue processing and sectioning, H&E staining of the 5 µm mouse skin sections was carried as detailed earlier [2]. Apoptotic cells were identified by TUNEL staining using the DeadEnd Colorimetric TUNEL system (Promega, Madison, WI) following a modified version of the manufacturer's protocol as detailed earlier [2]. The epidermal thickness (µm) and the brown TUNEL positive cells were quantified in 10 randomly selected fields at 400× magnification under a Zeiss Axioscop 2 microscope using Axiovision Rel 4.5 software (×400 magnification; Carl Zeiss Inc., Germany).

### Measurement of myeloperoxidase (MPO) activity

MPO activity was measured employing a kit from Cell Technology (Mountain View, CA) as published earlier [2], [9]. In brief, lysates were prepared from each exposed and treated clean skin tissue (∼100 mg), protein concentration was determined and reaction mixture prepared as detailed earlier [2]. 50 µL of reaction mixture and prepared sample (50 µg protein) or MPO standards were added in 96 well plates. After 1 h incubation at RT in the dark, fluorescence was measured at 530 nm excitation and 590 nm emission wavelengths. The blank control readings were subtracted from all the sample readings. The MPO activity was determined as mU/mL protein using the MPO standard curve.

### Preparation of total cell or tissue lysates and western blot analysis

At the end of desired exposures and treatments, total cell and tissue lysates were prepared in denaturing buffer, and protein content measured by Lowry's method as reported earlier [6], [26], [31]. Equal amounts of protein (60 or 80 µg) were denatured in sample buffer, and resolved on Tris-glycine gel (8–16%) by SDS, transferred to nitrocellulose membranes and blocked for 1 h with 5% nonfat milk/Odyssey blocking buffer. Membranes were incubated with the appropriate concentrations of primary antibodies O/N at 4°C, and then incubated with HRP conjugated/IRDye® 680LT conjugated secondary antibodies, and subjected to chemiluminescent detection (Amersham, Piscataway, NJ) or visualized using Odyssey™ Infrared Imager (LI-COR Biosciences, Lincoln, NE). Membranes were stripped and reprobed with anti-β-actin antibody (Sigma, St Louis, MO) for loading control. Primary antibodies used were H2AX.ser139, p53ser15, total p53, MMP-9 (Cell Signaling Technology, Beverly, MA); iNOS (Abcam Inc., Cambridge, MA), and anti-5,5-dimethyl-2-(8-octanoic acid)-1-pyrolline N-oxide (DMPO) nitrone polyclonal antiserum and COX-2 (Cayman Chemical Company, Ann Harbor, MI). Anti-4-hydroxynonenal (4-HNE) rabbit polyclonal antibody was kind gift from Dr. Dennis Petersen (University of Colorado Denver, USA). Secondary antibodies used were anti-rabbit IgG (Cell Signaling Technology, Beverly, MA), anti-mouse IgG (GE Healthcare Biosciences, Pittsburgh, PA) and IRDye® 680LT goat anti-mouse secondary antibody (LI-COR Biosciences, Lincoln, NE).

### COX-2 Immunoassay

COX-2 immunoassay was conduced using the ELISA kit from Antibodies-Online Inc. (Atlanta, GA) according to the manufacturer's protocol using equal amounts of protein (20 µg) from the exposed and treated skin tissue lysates. The COX-2 in the samples was measured spectrophotometrically at a wavelength of 450 nm and the COX-2 (pg/mL) concentration in the samples was determined by comparing optical density of the samples to the standard curve.

### Electrophoretic Mobility Shift Assay (EMSA)

Nuclear lysates from the exposed and treated skin tissue samples were prepared after homogenizing the tissue, protein estimated, and EMSA was carried out to determine the DNA binding activity of AP-1 and NF-κB as described previously [12], [32], [33]. Briefly, AP-1 and NF-κB consensus oligonucleotides (3.5 pmol; Santa Cruz Biotechnology) were radiolabeled with [γ-32P]ATP in the presence of T4 polynucleotide kinase in 10× kinase buffer according to the manufacturer's protocol (Promega, Madison, WI, USA). G-25 Sephadex column was used to separate labeled probe from free [γ-32P] ATP. For EMSA, nuclear extract was first incubated with 5× gel shift binding buffer and then with AP-1 and NF-κB probe (^32^P-labeled) for 20 min at 37°C. For super shift and competition assays, nuclear extracts were incubated with anti- cFos, -cJun, -p65 and -p50 antibodies and unlabeled-oligos and then labeled AP-1 and NF-κB oligos were added, respectively. The DNA-protein complexes and free [^32^P]-oligonucleotides were separated on 6% native polyacrylamide gel in EMSA-buffer by electrophoresis at 150 V/40 mA for 1 h at 25°C, gels dried and autoradiography performed.

### Immunohistochemistry

Following the desired exposures and treatments, immunohistochemistry (IHC) of skin sections was carried out as reported earlier [2], [9], [13]. Briefly, following the desired exposures and treatments for 24 h, 5 µm skin sections were incubated with mouse monoclonal anti-8-oxo-2-deoxyguanosine (8-OHdG; JalCA, Japan) antibody in PBS O/N at 4°C in humidity chamber after processing, antigen retrieval and blocking of the endogenous peroxide activity was performed as reported earlier [2]. The N-Universal negative control rabbit IgG antibody (DAKO, Carpienteria, CA) was used as a negative control. After washing in PBS, sections were incubated with the appropriate biotinylated secondary antibody for 1 h, incubated with HRP-conjugated streptavidin (DAKO) for 30 min and incubated in DAB for 5 min. Sections were counterstained with hematoxylin for 2 min followed by dehydration and mounted for microscopic observation. The brown-colored DAB positive nuclei were counted in 10 randomly selected fields (×400 magnification).

### Autoradiogram scanning, densitometric and Statistical Analyses

The immunoblots were scanned using Adobe Photoshop 6.0 (Adobe Systems, Inc., San Jose, CA)/or the Odyssey™ Infrared Imager. The densitometric analysis of the immunoblots/autoradiograms was carried out by measuring the integrated density using the Scion Image Program (NIH, Bethesda, MD). The statistical analyses were carried out using SigmaStat (software version 2.03, Jandal Scientific Corp., San Raphael, CA). Data are expressed as mean ± SEM and analyzed via one way ANOVA, followed by the Bonferroni or Tukey t-test for multiple comparisons. P values of <0.05 were considered statistically significant.

## Results

### Silibinin reverses CEES-induced decrease in cell viability in skin cells

Recently we showed that a decrease in cell viability is the major consequence of CEES exposure [6], and therefore first we assessed the effect of silibinin post-treatment on this parameter by MTT assay, where various silibinin concentrations and post-CEES injury time-points were analyzed for silibinin efficacy. Out of these screening studies, we found that silibinin treatment at 10 µM concentration and 30 min after CEES exposure for 24 h (or 48 h in case of NHEK) results in 29, 51 and 67% (p<0.05) reversal in CEES-induced decrease in cell viability in mouse epidermal JB6 cells, NHEK and mouse dermal fibroblasts, respectively (Fig. 1B, C and D).

### Silibinin reverses CEES-induced decrease in live cell number, and increase in apoptotic and necrotic cell death in skin cells

**Figure 2:**
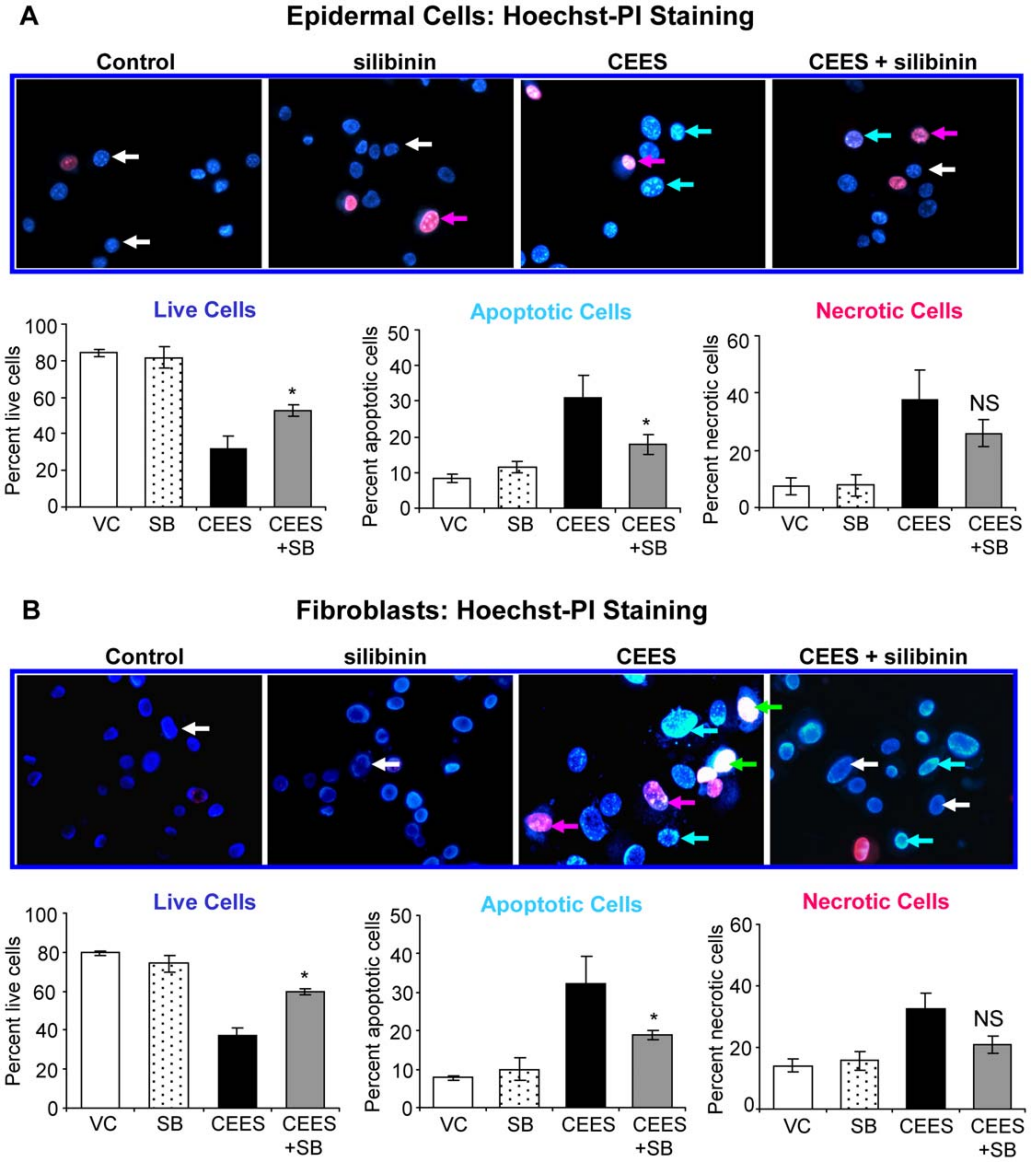
Silibinin reverses CEES-induced decrease in live cell number, and increase in apoptotic and necrotic cell death in skin cells. Mouse epidermal JB6 cells (**A**) and fibroblasts (**B**) were seeded (120,000 cells/plate) and grown overnight in 60 mm petri dishes. The cells were then exposed to DMSO or 0.35 mM CEES in DMSO, or treated with either 10 µM silibinin alone or with silibinin following 30 min CEES exposure for 24 h. At the end of each treatment time, cells were collected, washed twice with 1× PBS and stained with Hoechst-PI dye at a ratio of 3∶1 as detailed under Materials and Methods. The cells were counted manually under a fluorescence microscope in at least ten random fields and percent live, necrotic and apoptotic cells were calculated as detailed under Materials and Methods. Pictures were taken at 400× magnification, and the effect of CEES and silibinin treatments are shown in the representative pictures on top of the graphs showing the quantified data. Data shown are mean ± SEM of three independent samples. *, p<0.05 as compared to CEES exposed group. White arrows, live cells-blue fluorescence and normal structure; blue arrows, early apoptotic cells- bright blue florescence and enlarged with condensed or fragmented chromatin; green arrows, late apoptotic cells- bright red (pink to white) fluorescence in the centre due to condensed chromatin; red arrows, necrotic cells-bright red fluorescence and enlarged; VC, vehicle (DMSO) control; SB, silibinin; CEES+SB, silibinin treatment 30 min after CEES exposure; NS, not significant.

Since silibinin reversed CEES-induced decrease in cell viability, next we examined its overall effect on CEES-induced apoptotic and necrotic cell populations together with a decrease in live cells [6], [29]. Because silibinin demonstrated therapeutic efficacy in attenuating CEES-induced decrease in cell viability in mouse and human epidermal cell lines (Fig. 1B and C), further studies were performed only in mouse epidermal cells and fibroblasts as they were easy to handle and had more growth capacity. As shown in Fig. 2, silibinin treatment post-CEES injury resulted in 40 and 53% (p<0.05) reversal in CEES-induced loss of viability, 57 and 54% (p<0.05) reversal in CEES-induced apoptotic cell death, and 39 and 63% reversal in CEES-induced necrotic cell death, in JB6 cells (A) and fibroblasts (B), respectively..

### Silibinin reverses CEES-induced DNA damage in skin cells

**Figure 3:**
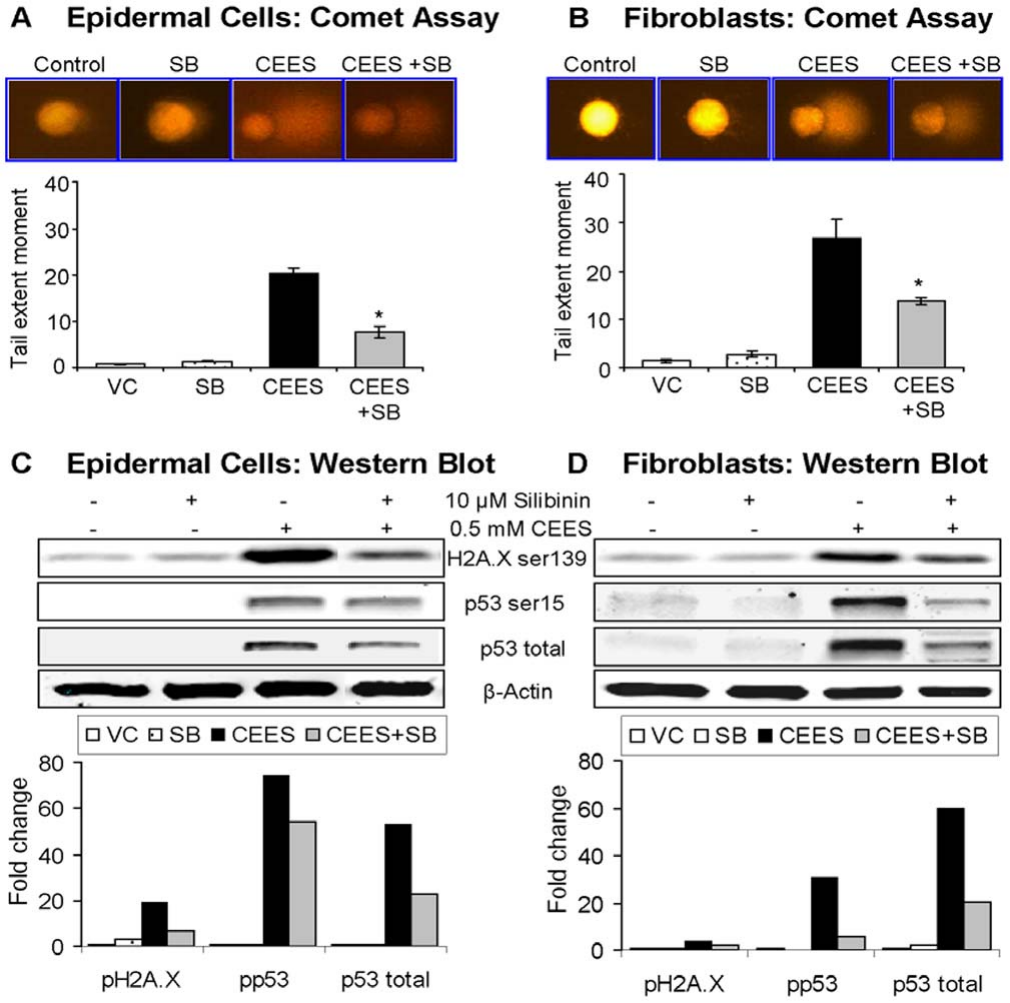
Silibinin reverses CEES-induced DNA damage in skin cells. Mouse epidermal JB6 cells (**A**) and fibroblasts (**B**) were seeded (120,000 cells/plate) and grown overnight in 60 mm petri dishes. The cells were then exposed to DMSO or 0.5 mM CEES in DMSO, or treated with either 10 µM silibinin alone or with silibinin following 30 min CEES exposure for 1 h. DNA damage in cells was measured using single cell gel electrophoresis (SCGE) or alkaline comet assay as detailed under materials and Methods. Damaged DNA seen in the form of comets [as seen in the representative pictures of epidermal cells (**A**) and fibroblasts (**B**) on top of graphical presentation of the quantified data in each panel] was scored and images were captured as described under Materials and Methods section. Data are presented as the tail extent moment (TEM; product of tail length and percentage tail DNA) and are mean ± SEM of three independent samples. *, p<0.05 as compared to CEES exposed group. DNA damage was also assessed via the detection of DNA damage markers H2A.X and p53 using western immunoblotting (**C and D**). Mouse epidermal JB6 cells (**C**) and dermal fibroblasts (**D**) grown over night in 100 mm plates were collected after 4 h of DMSO or 0.5 mM CEES exposure, or treatment with either 10 µM silibinin alone or with silibinin following 30 min CEES exposure, and cell lysates prepared in the lysis buffer as detailed under the Materials and Methods. The protein lysate was subjected to SDS-PAGE followed by immunoblotting using antibodies for H2A.X ser139 and p53 ser15 phosphorylations, and total levels of p53, and visualized and scanned using the Odyssey™ Infrared Imager. Protein loading was checked by stripping and re-probing the membranes with β-actin antibody and the results obtained were quantified by densitometric analysis of the immunoblots.

Cytotoxic effects of CEES are associated with its DNA damaging properties [6], [29], [34], and recently we have shown CEES-induced DNA damage in the form of increased TEM indicating damaged DNA in the cell (comet assay), H2A.X ser139 and p53 ser15 phosphorylation, and p53 protein accumulation (western immunoblotting) in both JB6 cells and fibroblasts [29]. Accordingly, next we examined the rescue efficacy of silibinin on CEES-induced DNA damage employing above established biomarkers. As shown in representative fluorescence micrographs of DNA comets and their quantification, silibinin post-treatment caused a 52 and 64% reversal (p<0.05) in CEES-induced TEM in both JB6 cells and fibroblasts, respectively (Fig. 3A and B). Similarly, as shown by immunoblots and their quantifications, silibinin also caused a 68, 26 and 57% (JB6 cells) and 63, 85 and 67% (fibroblasts) reversal in CEES-induced H2A.X and p53 phosphorylation and p53 accumulation, respectively (Fig. 3C and D).

### Silibinin reverses CEES-induced oxidative stress in skin cells

**Figure 4:**
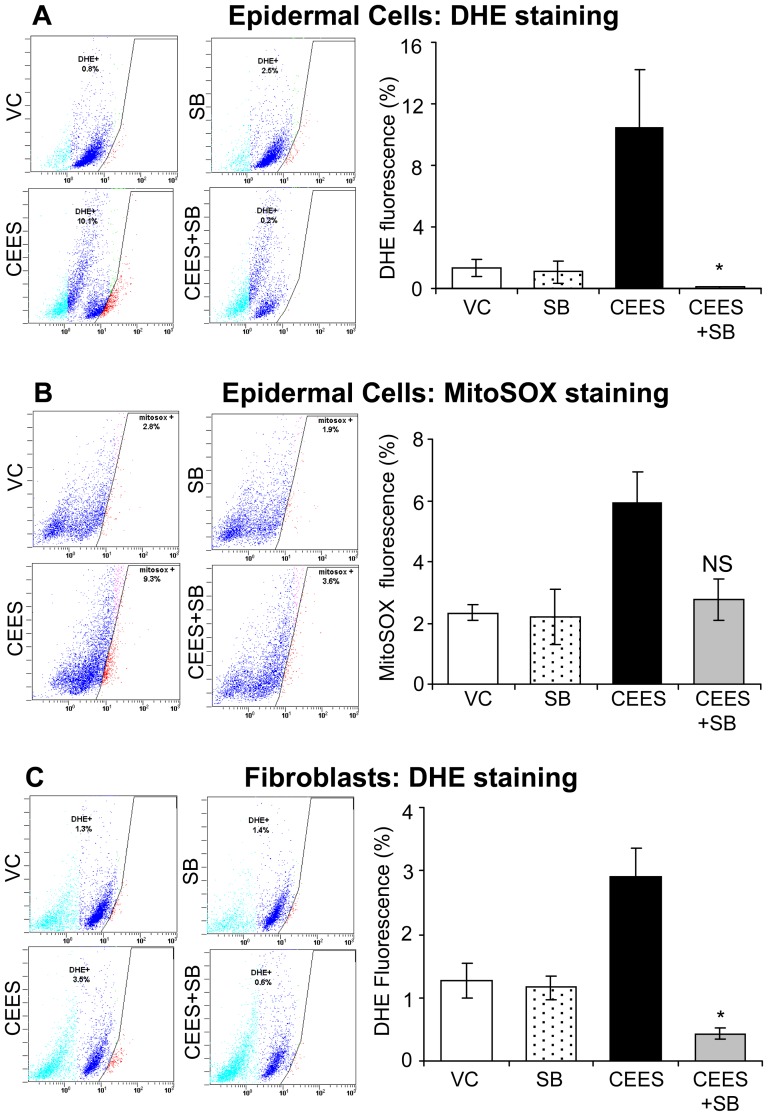
Silibinin reverses CEES-induced oxidative stress in skin cells. Mouse epidermal JB6 cells (**A and B**) and fibroblasts (**C**) were seeded (120,000 cells/plate) and grown overnight in 60 mm petri dishes. The cells were then exposed to DMSO or 0.5 mM CEES in DMSO, or treated with either 10 µM silibinin alone or with silibinin following 30 min CEES exposure for 4–6 h. The cells were then incubated for 30 min or 1 h with DHE (**A and C**) or MitoSOX Red (**B**), respectively, and the live cell fluorescence was determined using flowcytometry as described under Materials and Methods. Representative pictures of the flow cytograms of the epidermal JB6 cells (**A and B**) and dermal fibroblasts (**C**) are shown on the left side of the graphical presentation of the quantitative data. Data shown are mean ± SEM of three independent samples. *, p<0.05 as compared to CEES exposed group. VC, vehicle (DMSO) control; SB, silibinin; CEES+SB, silibinin treatment 30 min after CEES exposure; NS, not significant.

Our recent studies have shown a depletion of GSH and an increase in oxidative stress (mainly O_2_^−^ production) by CEES in both skin cells in culture and mouse skin, which have been implicated in vesicant-induced DNA damage [19], [29]. Accordingly, we next assessed the efficacy of silibinin in reversing CEES-induced cellular and mitochondrial oxidative stress, by using DHE and MitoSOX Red, respectively, which detect O_2_^−^ levels, as reported earlier [29]. In JB6 cells, silibinin (10 µM) treatment, 30 min following CEES exposure, resulted in a complete and 85% reversal (p<0.05) in CEES-caused cellular and mitochondrial O_2_^−^ levels, respectively (Fig. 4A and B). Similar silibinin treatment also showed a complete reversal in CEES-induced cellular O_2_^−^ levels in fibroblasts (Fig. 4C).

### Silibinin reverses CEES-induced skin injury attributes in mouse skin

**Figure 5:**
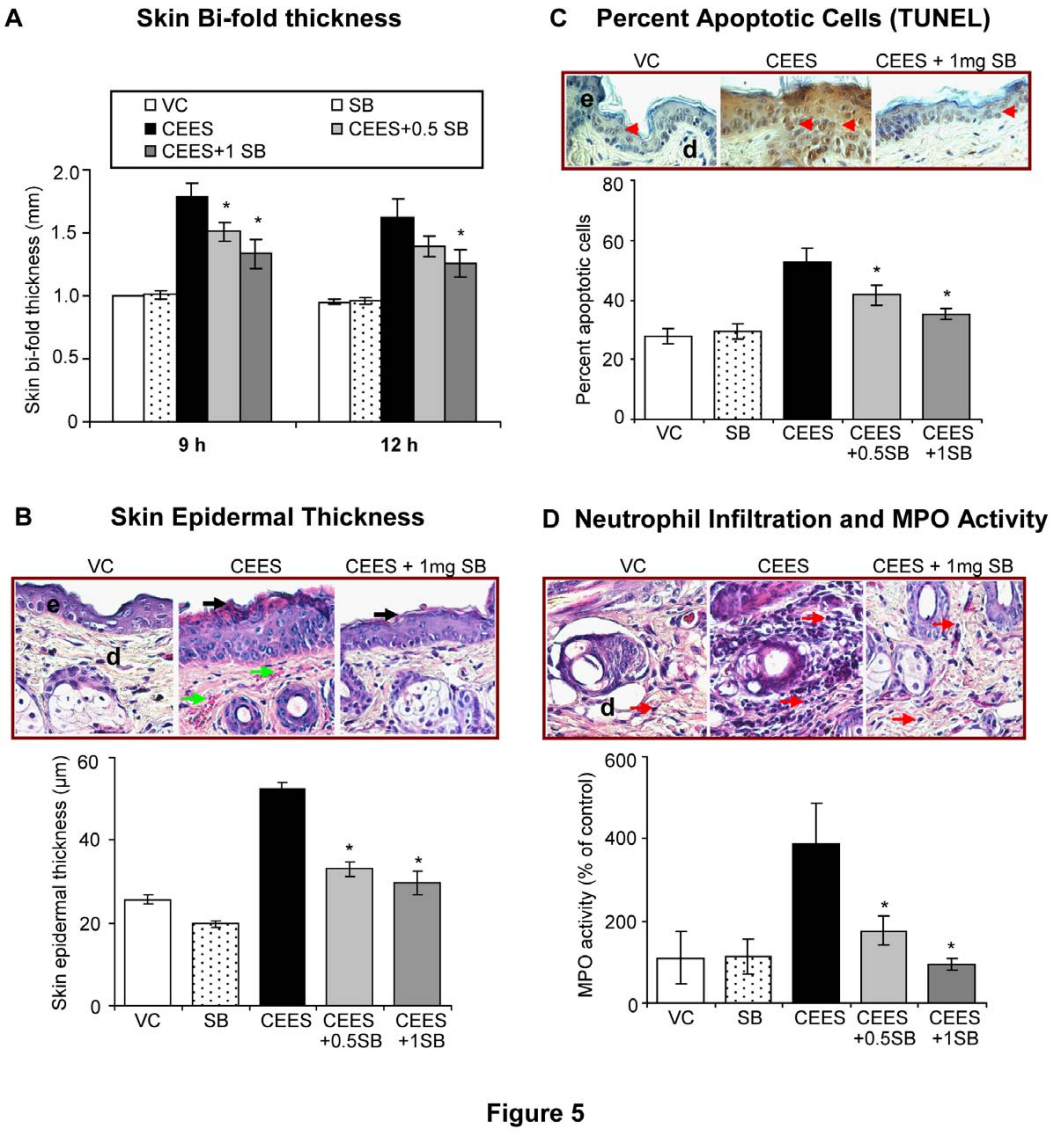
Silibinin reverses CEES-induced skin injury and inflammatory biomarkers in SKH-1 hairless mouse skin. Dorsal skin of mice was exposed topically to either 200 µL of acetone or CEES (2 mg) in 200 µL acetone, or treated with either silibinin alone or with 0.5 or 1 mg silibinin 30 min after CEES exposure. After 9, 12 and 24 h of the indicated treatments, skin bi-fold thickness was measured using a digital caliper (**A**) as detailed under Materials and Methods. Mice were sacrificed and dorsal skin tissue samples were collected at 24 h following the above exposures and treatment. The skin sections (5 µM) were processed, H&E stained and analyzed for histopathological changes [representative pictures show changes in the epidermal thickness, epidermal necrosis, increase in blood vessels (**A**) and neutrophil infiltration (**B**)] and quantified for epidermal thickness (**B**; 400× magnification). Apototic cell death was assessed and quantified in the skin sections subjected to DeadEnd Colorimetric TUNEL assay (**C**) as detailed under Materials and Methods. The epidermal thickness (µm) and the apoptotic index (positive cells ×100/total number of cells) were calculated after their quantification in 10 randomly selected fields at 400× magnification under a microscope. To determine the increase in neutrophil infiltration observed in the H&E stained skin sections (representative pictures of H&E stained dermis demonstrated an increase in the neutrophil infiltration **D**, top panels), treated skin tissue samples were used for lysate preparation and MPO activity was determined by a fluorescence assay (**D**; bottom graph) as detailed under Materials and Methods. Data presented are mean ± SEM of 5 animals in each treatment group. *, p<0.05 as compared to CEES exposed group. VC, vehicle (acetone); SB, silibinin; CEES+0.5 SB, 0.5 mg silibinin treatment 30 min after CEES exposure; CEES+1SB, 1 mg silibinin treatment 30 min after CEES exposure; e, epidermis; d, dermis; black arrow, epidermal necrosis; green arrows, blood vessels; red arrowheads, TUNEL positive brown cells; red arrows, neutrophils.

Our recent findings in SKH-1 hairless mouse have established skin bi-fold thickness, epidermal thickness, apoptotic cell death, and MPO activity (indicating neutrophil infiltration) as important quantifiable biomarkers of CEES-induced skin injuries suggesting their usefulness in rescue agent efficacy studies [2], [9]. In an effort to determine its therapeutic efficacy in skin cells *in vivo*, we applied silibinin (0.5 and 1 mg) topically on to the mice 30 min following CEES (2 mg) exposure, and skin tissues were analyzed for the above-established biomarkers. In all the study endpoints, 1 mg silibinin was more effective as compared to 0.5 mg silibinin in attenuating the CEES-induced skin injury responses (Fig. 5). Treatment with silibinin (1 mg) caused 62% (p<0.05) reversal in CEES-induced increase in skin bi-fold thickness at both 9 and 12 h, respectively (Fig. 5A). At 24 h after CEES exposure, silibinin treatment did not show a significant reversal in CEES-induced skin bi-fold thickness (data not shown). However, at this time point, silibinin (1 mg) treatment was more effective in causing 85%, 70% and complete reversal (p<0.05) in CEES-induced increases in epidermal thickness, apoptotic cell death and MPO activity, respectively (Fig. 5B–D). Further analyses of H&E stained skin tissue sections also showed that silibinin reversed CEES-induced epidermal desquamation and necrosis (black arrows; Fig. 5B), increase in the blood vessels (green arrows; Fig. 5B), and neutrophil infiltration (red arrows; Fig. 5D).

### Silibinin reverses CEES-caused increase in inflammatory and vesicating mediators, and activation of NF-κB and AP-1 transcription factors in mouse skin

**Figure 6:**
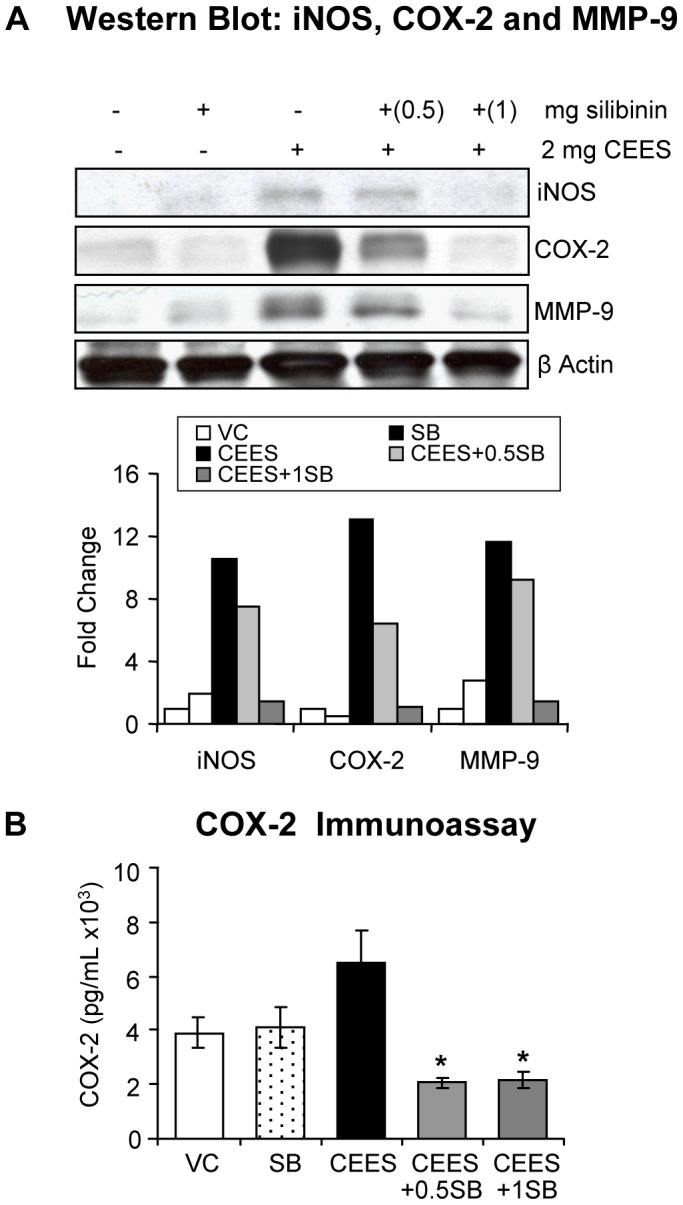
Silibinin reverses CEES-induced increase in the expression of inflammatory and vesicating mediators in SKH-1 hairless mouse skin. Dorsal skin of mice was exposed topically to either 200 µL of acetone or CEES (2 mg) in 200 µL acetone, or treated with either silibinin alone or with 0.5/1 mg silibinin 30 min after CEES exposure. Mice were sacrificed and dorsal skin tissue samples were collected and snap frozen in liquid nitrogen at 24 h following the above exposures and treatment. Skin tissue lysates were prepared and about 80 µg of protein lysate per sample was loaded and subjected to SDS-PAGE followed by transfer to membranes as detailed under Materials and Methods. The membranes were probed for iNOS, COX-2 and MMP-9 and subjected to chemiluminescent detection (**A**) according to the details under Materials and Methods. Protein loading was checked by stripping and re-probing the membranes with β-actin antibody and the results obtained were quantified by densitometric analysis of the immunoblots (**A**). Following the above treatments cell lysates were prepared and 20 µg protein was used for measuring the COX-2 concentration in the skin tissue (**B**), which was conduced using an ELISA kit following the manufacturer's protocol. Data presented are mean ± SEM (n = 3); *, p<0.05 as compared to CEES exposed group; VC, vehicle (acetone); SB, silibinin; CEES+0.5SB, 0.5 mg silibinin treatment 30 min after CEES exposure; CEES+1SB, 1 mg silibinin treatment 30 min after CEES exposure.

Our recent studies have also shown that CEES exposure causes the activation of signal transduction pathways and transcription factors resulting in an increased expression of inflammatory and proteolytic mediators such as COX-2, iNOS and MMP-9, which leads to both inflammation and vesication in SKH-1 mouse skin [12], [13]. Since silibinin is known to target these inflammatory mediators and related pathways [33], we next assessed its effect on CEES-caused induction of these inflammatory and vesication/proteolytic regulators. Silibinin (1 mg) treatment 30 min following CEES exposure caused almost complete (95–99%) reversal in CEES-induced increase in the expression of COX-2, iNOS and MMP-9 (Fig. 6A). Since COX-2 derived prostaglandins are key mediators of inflammation, facilitating the influx of inflammatory cells, we also assessed the effects of silibinin on CEES-mediated increases in cellular COX-2 concentration (pg/mL). COX-2 immunoassay showed that silibinin treatment resulted in complete reversal of CEES-induced increases in COX-2 concentrations (pg/mL) in mouse skin tissue (Fig. 6B).

**Figure 7:**
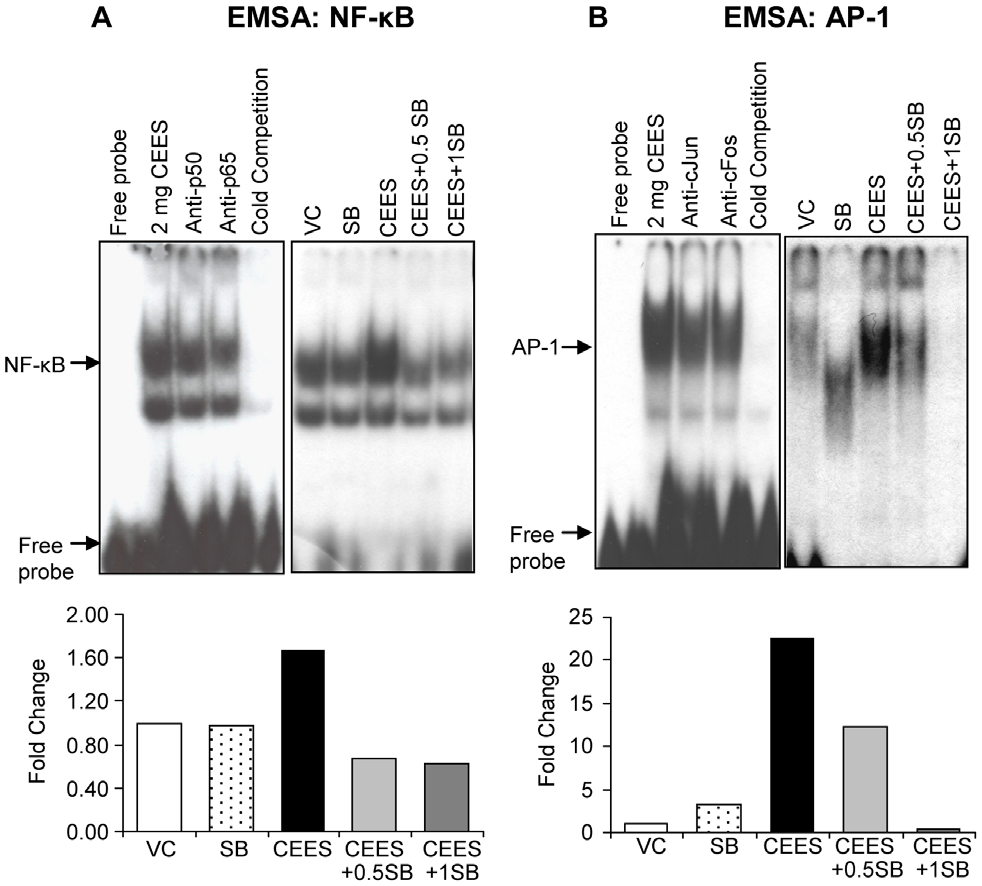
Silibinin reverses CEES-induced activation of NF-κB and AP-1 transcription factors in SKH-1 hairless mouse skin. Dorsal skin of mice was exposed topically to either 200 µL of acetone or CEES (2 mg) in 200 µL acetone, or treated with either silibinin alone or with 0.5/1 mg silibinin 30 min after CEES exposure. Mice were sacrificed and dorsal skin tissue samples were collected and snap frozen in liquid nitrogen at 24 h following the above exposures and treatment. Nuclear lysates were prepared from the skin tissue and analyzed for NF-κB (**A**) and AP-1 (**B**) DNA binding activity by EMSA as detailed under Materials and Methods. Nuclear extract from CEES exposed skin tissue samples were used for cold competition with cold NF-κB (**A**) or AP-1 (**B**) consensus oligo, and super shift assay was performed as detailed under Materials and Methods. The results obtained were quantified by densitometric analysis. VC, vehicle (acetone); SB, silibinin; CEES+0.5SB, 0.5 mg silibinin treatment 30 min after CEES exposure; CEES+1SB, 1 mg silibinin treatment 30 min after CEES exposure.

Our recent study and other published reports show that CEES-induced inflammatory responses involve the activation of transcription factors AP-1 and NF-κB, which influences the induction of MMPs, iNOS and COX-2 levels and that silibinin targets them in its anti-inflammatory activity [12], [35], [36]. Accordingly, these were next analyzed where EMSA results from the nuclear extracts of the tissues showed that silibinin treatment at both 0.5 and 1 mg doses caused a complete reversal of NF-κB activation by CEES (Fig. 7A). Regarding AP-1, whereas 0.5 mg silibinin dose caused a strong reversal, 1 mg dose caused a complete reversal of CEES-induced AP-1 activation (Fig. 7B). As reported in our earlier studies, CEES-exposed skin showed activation of AP-1 family proteins (c-Fos and c-Jun) and NF-κB (p50 and p65) [12], providing further evidence that silibinin targets NF-κB and AP-1 transcription factors and related pathways.

### Silibinin reverses CEES-induced lipid peroxidation, DMPO nitrone protein adduct formation and DNA oxidation in mouse skin

**Figure 8:**
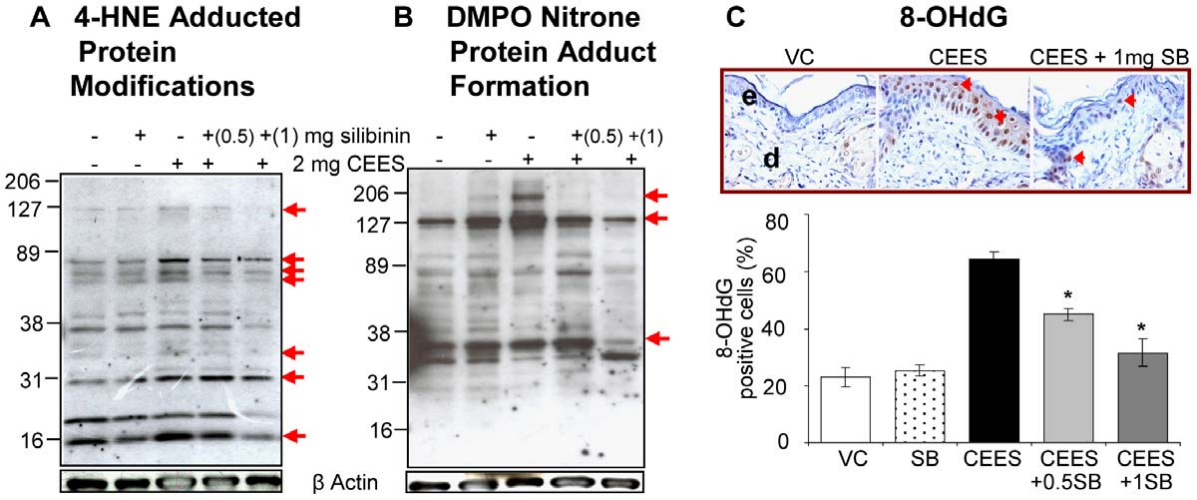
Silibinin reverses CEES-induced lipid peroxidation, protein adduct formation and DNA oxidation in SKH-1 hairless mouse skin. Dorsal skin of mice was exposed topically to either 200 µL of acetone or CEES (2 mg) in 200 µL acetone, or treated with either silibinin alone or with 0.5/1 mg silibinin 30 min after CEES exposure. Mice were sacrificed and dorsal skin tissue samples were collected and snap frozen in liquid nitrogen at 24 h following the above exposures and treatment. Skin tissue lysates were prepared and protein lysate was loaded and subjected to SDS-PAGE followed by immunoblotting using antibodies for 4-HNE (**A**) and DMPO (**B**) as detailed under Materials and Methods. The membranes were subjected to chemiluminescent detection (**A and B**) according to details under Materials and Methods. Protein loading was checked by stripping and re-probing the membranes with β-actin antibody. Following the above mentioned exposures and treatment for 24 h, mice were sacrificed and dorsal skin tissue samples were collected and fixed in formalin as detailed under Materials and Methods. The 5 µm skin sections after processing were subjected to IHC for 8-OHdG (**C**) as detailed under the Materials and Methods section. The brown colored DAB positive nuclei were 8-OHdG positive (as shown in the representative pictures; **C**) and were counted in 10 randomly selected fields (400× magnification). Data presented are mean ± SEM of 5 animals in each treatment group. *, p<0.05 as compared to CEES exposed group. VC, vehicle (acetone); SB, silibinin; CEES+0.5SB, 0.5 mg silibinin treatment 30 min after CEES exposure; CEES+1SB, 1 mg silibinin treatment 30 min after CEES exposure; e, epidermis; d, dermis; red arrowheads, 8-OHdG positive brown cells.

Our previous findings and those of others suggest that oxidative stress could be an initiating event after vesicant exposure, activating complex signal transduction pathways leading to the induction of inflammatory mediators, with secondary inflammatory and vesicating responses [6], [12]–[14], [19]. Therefore, we next determined if silibinin reverses this key event, which might in turn attenuate all related downstream responses. Employing established biomarkers of oxidative stress, such as 4-HNE (lipid peroxidation), DMPO (nitrone protein adduct formation) and 8-OHdG formation (oxidative DNA damage) from our earlier studies [12], we assessed the efficacy of silibinin in reversing CEES-induced oxidative stress (Fig. 8). Similar to the above results, 1 mg silibinin was more effective as compared to 0.5 mg silibinin in reversing CEES-induced 4-HNE protein adduct modifications (Fig. 8A), DMPO nitrone protein adduct formation (Fig. 8B), and 8-OHdG levels (78%; p<0.05; Fig. 8C) in mouse skin, suggesting this to be a potential upstream event for the observed silibinin efficacy in reversing CEES-induced skin injury biomarkers in SKH-1 hairless mice.

## Discussion

The threat of warfare mustard vesicants' exploitation also as potential terrorist agents poses challenges to the research community to develop effective therapies against vesicant-related injuries [18], [37]. Skin injuries by HD can take several months to heal and require long-term medical management. However, the best remedy currently available is decontamination along with supportive treatment to relieve pain, prevent infection and promote healing [38], [39]. Exposure to HD triggers an array of complex signal transduction pathways, suggesting the need for multifunctional/pleiotropic agents or combination therapies to treat skin injuries by this vesicant [6], [12], [13], [16], [17], [29], [40], [41]. Findings in the present study demonstrated the strong therapeutic efficacy of natural flavanone silibinin in attenuating HD analog CEES-induced cutaneous toxic responses. These studies further give insight into the antioxidant and potential pleiotropic mechanisms of silibinin in reversing toxic effects of CEES exposure.

The current study was carried out using skin toxicity models of CEES, a less toxic monofunctional alkylating analog of HD that only has the ability to form adducts instead of cross-links with biological molecules [6], [42], [43]. However, since CEES has similar chemical properties and toxic effects as HD, this study supports earlier reports of its use as a practical substitute to study molecular effects of injury to screen treatment agents in the laboratory settings [2], [6], [43]. Once identified, therapeutic agents need to be confirmed for efficacy in bifunctional alkylating vesicant skin injury model. Constantly dividing basal epidermal keratinocytes are reported to be the primary targets of the vesicating agents. However, due to the presence of dermal injury and infiltration of inflammatory molecules, toxic effects on fibroblasts are also evident, which contribute to vesication by HD [5], [7], [29], [44]–[47]. Therefore, efficacy studies herein were carried out in both skin epidermal cells and dermal fibroblasts employing biomarkers established in our earlier reported studies [6], [29]. The observed therapeutic efficacy of silibinin in cell culture studies was then advanced in our *in vivo* studies.

**Figure 9:**
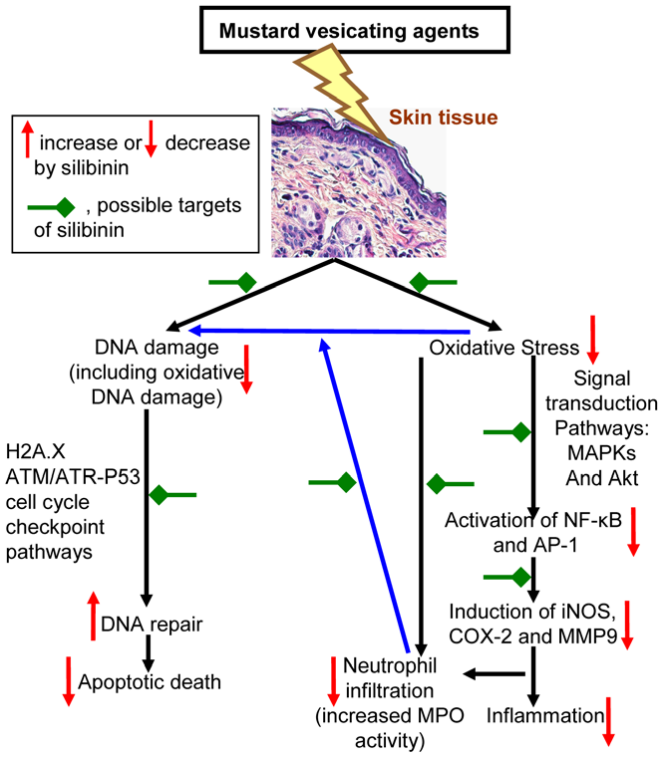
Schematic of possible therapeutic targets for silibinin in CEES-induced skin injury pathways identified in our studies. Possible targets of silibinin (red green arrows); increase or decrease by silibinin (up or down arrows, respectively).

Vesicating agents are strong alkylating agents, and their interaction with cellular thiols, especially GSH, leads to accumulation of endogenous ROS, resulting in lipid peroxidation, protein adduct formation and DNA damage which can subsequently activate an array of signaling pathways [10]–[14], [19]. Knowledge of these complex mechanisms of action of vesicating agents is important to develop effective therapies. Accordingly, our recent studies in skin cells and mouse skin tissue have revealed that HD analog CEES induces oxidative stress, DNA damage-related cell cycle checkpoint signaling, activation of signaling pathways including MAPKs and Akt, and subsequent activation of transcription factors AP-1 and NF-κB, resulting in the induction of inflammatory (COX-2, iNOS) and vesicating mediators (MMP-9) [6], [12], [13], [29]. The consequences of CEES exposure on skin observed in our studies and reported by others fairly resemble those of ultraviolet B (UVB) radiation, where silibinin is reported to be an effective chemopreventive agent [21], [22], [25], [26], [28]. Silibinin is reported to target p53, Cip1/p21 and other cell cycle-regulatory molecules to prevent UVB-induced skin carcinogenesis, in part by controlling apoptotic cell death and DNA damage repair [22]. Upon exposure to vesicating agents, p53 is phosphorylated leading to its stabilization and accumulation. Ultimately, there is an increase in the nuclear level of p53 with the formation of a homotetrameric complex acting as a transcriptional suppressor or activator [22]. In the current study, the ability of silibinin to attenuate CEES-induced apoptotic cell death, p53 accumulation and ser15 phosphorylation demonstrates its mode of action on p53-dependent apoptosis, cell cycle arrest and DNA damage repair. Silibinin treatment 30 min after CEES-exposure also reversed CEES-induced H2A.X ser139 phosphorylation, which is an indicator of the cellular response to DNA damage, mainly the DSB's. Similarly, silibinin treatment also reversed CEES-induced cellular and mitochondrial O_2_^−^ levels which are reported to be important events leading to oxidative DNA damage [29], [43]. Therefore, oxidative stress could be a target of silibinin in the regulation of CEES-induced DNA damage. However, silibinin could also directly influence p53 and H2A.X in cell cycle regulation, apoptosis and DNA repair, suggesting their importance as mediators of therapeutic efficacy by silibinin against CEES-induced skin injuries (Fig. 9).

Silibinin is also known to target an array of cellular signaling pathways and molecules such as MAPKs, Akt, AP-1, NF-κB, COX-2, iNOS and MMP-9, which are known to play a key role in skin tissue damage and inflammatory responses by both physical and chemical carcinogens [21], [24]. Consistent with these, in the present study, silibinin treatment 30 min after CEES-exposure significantly reversed CEES-induced a) inflammatory responses including increase in skin bi-fold and epidermal thickness, apoptotic cell death, neutrophil infiltration and MPO activity; b) induction of biochemical mediators: iNOS, COX-2 and MMP-9; c) activation of NF-κB and AP-1; and d) oxidative stress response: lipid peroxidation, protein oxidation and oxidative DNA damage in mouse skin. Diverse cell-injury stimuli, including oxidative stress, activate MAPKs and Akt pathways, which then activate transcription factors AP-1 and NF-κB are central players in regulating inflammatory and vesicant responses by facilitating the transcription of COX-2, iNOS and MMPs [12]. Our recent studies have reported CEES-induced oxidative stress-mediated activation of these pathways, leading to skin inflammatory and injury responses [12]. Therefore, the results shown here indicate that oxidative stress could be a target of therapeutic efficacy of silibinin against CEES-induced skin injury (Fig. 9). Since an increase in MPO activity, indicating neutrophil infiltration, can also result in further increase of ROS generation, reversal in CEES-induced MPO activity by silibinin further suggests its strong antioxidant effect by scavenging ROS or its potential to up regulate cells' antioxidant potential (Fig. 9).

Although oxidative stress, inflammation and activation of proteases in vesicant-related skin injury are reported as major consequences of vesicant exposure; antioxidants, scavengers, anti-inflammatory drugs and protease inhibitors alone do not exhibit strong therapeutic effects [40]. This suggests that agents are needed with diverse activity towards multiple pathways including targeting the oxidative stress. Natural flavanone silibinin is reported to be a strong antioxidant, and it also possesses strong anti-inflammatory, anti-angiogenic, anti-metastatic, and DNA repair properties [21], [22], [24], [33].Whereas targeting induction of oxidative stress has been a major focus for development of effective rescue therapies for vesicants [14], [18], [20], current study shows that reversal of the toxic effects of CEES by silibinin could be due to its antioxidant as well as other pleiotropic properties which target multiple signaling pathways related to DNA damage, inflammation and vesication (Fig. 9). This study also supports further optimization of silibinin as a rescue agent in HD-skin injury models to allow its development as an effective novel therapeutic agent against vesicant-induced skin injury.
